# Racial Disparities in Cancer Stage at Diagnosis and Survival for Adolescents and Young Adults

**DOI:** 10.1001/jamanetworkopen.2024.30975

**Published:** 2024-08-30

**Authors:** Kekoa Taparra, Kaeo Kekumano, Ryan Benavente, Luke Roberto, Megan Gimmen, Ryan Shontell, Henrietta Cakobau, Neha Deo, Connor J. Kinslow, Allison Betof Warner, Curtiland Deville, Jaimie Z. Shing, Jacqueline B. Vo, Manali I. Patel, Erqi Pollom

**Affiliations:** 1 Department of Radiation Oncology, Stanford Medicine, Stanford, California; 2 Department of Stem Cell and Regenerative Biology, Harvard College, Cambridge, Massachusetts; 3 Harvard Medical School, Boston, Massachusetts; 4 Department of Emergency Medicine, UC Davis Medical Center, Sacramento, California; 5 Department of Native Hawaiian Health, University of Hawaiʻi John A. Burns School of Medicine, Honolulu; 6 Department of Cognitive Science, University of California, Berkeley; 7 Internal Medicine Division, Department of Medicine, Mass General Hospital, Boston, Massachusetts; 8 Department of Radiation Oncology, Columbia University, New York, New York; 9 Department of Medicine, Division of Oncology, Stanford Medicine, Stanford, California; 10 Department of Radiation Oncology and Molecular Radiation Sciences, Johns Hopkins University School of Medicine, Washington, DC; 11 Division of Cancer Epidemiology and Genetics, National Cancer Institute, Rockville, Maryland; 12 Radiation Epidemiology Branch, Division of Cancer Epidemiology, National Cancer Institute, Bethesda, Maryland; 13 Medical Services, Veterans Affairs Palo Alto Health Care, Palo Alto, California

## Abstract

**Importance:**

There are limited studies assessing stage at diagnosis and risk of death among all 5 federally defined races in the US among adolescent and young adult (AYA) patients with cancer.

**Objective:**

To identify racial disparities in stage at diagnosis and survival among AYA patients with cancer.

**Design, Setting, and Participants:**

This retrospective cohort study used data from a US national hospital-based oncology database on AYA patients, aged 15 to 39 years, with the 10 deadliest cancers among AYA patients who received a diagnosis from January 1, 2004, to December 31, 2017, with 6 months or more of follow-up. Analyses by race were categorized by the 5 federally defined races in the US: American Indian or Alaska Native, Asian, Black, Native Hawaiian or Other Pacific Islander, and non-Hispanic White (hereafter, *White*). White patients served as the majority reference group. Statistical analysis was performed from November 2022 to September 2023.

**Main Outcomes and Measures:**

The primary end points were late stage at diagnosis (logistic regression with adjusted odds ratios [AORs] and 95% CIs) and overall survival (log-rank tests and Cox proportional hazards regression with adjusted hazard ratios [AHRs] and 95% CIs).

**Results:**

A total of 291 899 AYA patients (median age, 33 years [IQR, 28-37 years]; 186 549 female patients [64%]; 189 812 [65%] with stage I or II cancers) were evaluated. The cohort included 1457 American Indian or Alaska Native patients (1%), 8412 Asian patients (3%), 40 851 Black patients (14%), 987 Native Hawaiian or Other Pacific Islander patients (0.3%), and 240 192 White patients (82%). Cancers included breast (n = 79 195 [27%]), lymphoma (n = 45 500 [16%]), melanoma (n = 36 724 [13%]), testis (n = 31 413 [11%]), central nervous system (n = 26 070 [9%]), colon or rectum (n = 22 545 [8%]), cervix (n = 20 923 [7%]), sarcoma (n = 14 951 [5%]), ovary (n = 8982 [3%]), and lung (n = 5596 [2%]). Risk of late-stage diagnosis was higher for Asian (AOR, 1.20; 95% CI, 1.14-1.26), Black (AOR, 1.40; 95% CI, 1.36-1.43), and Native Hawaiian or Other Pacific Islander (AOR, 1.34; 95% CI, 1.16-1.55) patients compared with White patients. Overall survival differed by race for all cancer sites, except cancers of the central nervous system and ovary. Risk of death was higher for American Indian or Alaska Native (AHR, 1.15; 95% CI, 1.02-1.30), Black (AHR, 1.22; 95% CI, 1.19-1.26), and Native Hawaiian or Other Pacific Islander (AHR, 1.25; 95% CI, 1.09-1.44) patients but lower for Asian patients (AHR, 0.90; 95% CI, 0.85-0.95) compared with White patients.

**Conclusions and Relevance:**

This cohort study of AYA patients suggests that stage at diagnosis and survival varied across races for the 10 deadliest AYA cancers. These results support the need for tailored interventions and informed public policy to achieve cancer care equity for all races.

## Key Points

**Question:**

Are there racial disparities in stage at diagnosis and survival among adolescent and young adult (AYA) patients with cancer?

**Findings:**

In this cohort study of 291 899 AYA patients, the risk of late-stage diagnosis was significantly higher for Asian, Black, and Native Hawaiian or Other Pacific Islander patients compared with White patients. However, compared with White patients, the risk of death was significantly higher for American Indian or Alaska Native, Black, and Native Hawaiian or Other Pacific Islander patients but lower for Asian patients.

**Meaning:**

This study suggests that racial disparities in cancer stage at diagnosis and survival exist among AYA patients when disaggregated according to federal guidelines, which has health policy and funding implications.

## Teaser

This cohort study examines racial disparities in stage at diagnosis and overall survival for the 10 deadliest cancers among adolescents and young adults.

## Introduction

Adolescent and young adult (AYA) patients with cancer, aged 15 to 39 years, are a unique population with distinct demographic characteristics, societal challenges, and health care needs.^1,2^ The incidence of cancer among AYA patients has increased, with approximately 90 000 new diagnoses in 2023, 8 times that of children (aged ≤14 years).^3,4,5^ Despite these trends, AYA cancers remain the least studied, having limited survival improvements relative to children and adults,^6^ with underdeveloped cancer care guidelines (eg, fertility services, clinical trial enrollment, and psychosocial support)^7,8,9,10^ and poor treatment efficacy among select AYA patients.^11^ Furthermore, advanced-stage cancers are increasing among AYA patients, with a disproportionate increase in metastatic sites, especially compared with older adults.^12^

Differences in AYA treatment access and clinical outcomes are influenced by socioeconomic status, insurance, race and ethnicity, and sex.^10,13,14,15^ Mortality disparities are well known among Black and Hispanic communities, even for cancers with favorable prognoses.^14^ However, Indigenous races, including American Indian or Alaska Native populations and Native Hawaiian or Other Pacific Islander populations, are often excluded from studies, resulting in poor understanding of their health outcomes.^16,17,18^ Emerging evidence demonstrates that Native Hawaiian or Other Pacific Islander populations have the highest age-standardized cancer mortality among patients aged 15 to 49 years in the US.^15^ As the incidence of AYA cancer increases, understanding racial disparities—when properly disaggregated according to federal standards—is vital.^19^ Thus, this study aimed to quantify differences in the risk of late-stage diagnosis and overall survival (OS) for the 10 deadliest AYA cancers by federally defined racial categories in the US.

## Methods

### Data Source and Patient Population

This retrospective cohort study was exempt from review and the informed consent process by the Stanford University institutional review board because the data were deidentified and publicly available. This study followed the Strengthening the Reporting of Observational Studies in Epidemiology (STROBE) reporting guideline.

Adolescent and young adult patients diagnosed between January 1, 2004, and December 31, 2017, were evaluated using the National Cancer Database (NCDB). The top 10 deadliest AYA solid malignant neoplasms were included, defined by the AYA Surveillance, Epidemiology, and End Results Program (age-adjusted death rates per 100 000 individuals): breast cancer, central nervous system (CNS) cancer, cervical cancer, colon or rectal cancer, lung or bronchial cancer, lymphoma, melanoma, ovarian cancer, sarcoma, and testicular cancer.^5^ Patients with 6 months or more of follow-up, a diagnostically confirmed malignant neoplasm, complete staging, and known vital status were included. Patients with unknown vital status, unknown follow-up, and no diagnostic confirmation of cancer were excluded. Analyses included the 5 federally defined self-reported races: American Indian or Alaska Native, Asian, Black, Native Hawaiian or Other Pacific Islander, and White.^20^ Hispanic ethnicity was mutually exclusive with race to prevent small sample sizes after ethnicity stratification, with the exception of the majority non-Hispanic White (hereafter, *White*) race.^21^

### Statistical Analysis

Statistical analysis was performed from November 2022 to September 2023. Racial differences in late stage at diagnosis (stage III or IV; CNS tumors grade III or IV) were assessed using logistic regressions and adjusted odds ratios (AORs) with 95% CIs, accounting for patient covariables and cancer characteristics. Covariates were based on NCDB definitions,^22^ as described previously,^21^ and included age (in years), distance to hospital (kilometers between patient zip code or city centroid and hospital), county-based rurality (metropolitan vs urban or rural), zip code–based income (above vs below median), zip code–based educational level (above vs below median county high school graduation rates), insurance status (private insurance, Medicaid or Medicare, uninsured, vs unknown), and comorbidity burden (Charlson-Deyo Comorbidity Index of 0-2 vs ≥3). Select covariates routinely used in NCDB studies, including facility type and location, are suppressed for patients aged 39 years or younger, so they were not included.^22^ Socioeconomic status variables were estimated based on census data. Income and education were dichotomized at the median: “higher income” or “more education” referred to household income adjusted for inflation or high school degree attainment above the median US county rates, respectively, as estimated from the 2016 American Community Survey.^22^ Cancer characteristics included stage (grade for CNS tumors), year of diagnosis, and receipt of treatment (surgery, chemotherapy, or radiotherapy at any point of treatment).

We assessed OS (time from diagnosis to death) using Kaplan-Meier estimates and log-rank tests. One-, 5-, and 10-year OS were calculated, considering the extended survivorship of AYA patients, which can span 5 or 6 decades.^23^ To identify the greatest relative gaps in 10-year OS, the absolute OS differences were calculated between the races with the highest and lowest 10-year OS rates for each cancer, acknowledging that the referent racial groups may vary across cancers depending on the race with the lowest survival. We evaluated differences in risk of death by race using multivariable Cox proportional hazards regression (adjusted hazard ratios [AHRs] with 95% CIs), adjusting for patient and cancer covariates.

Patients with missing data were included and reported accordingly. Models were checked for statistical assumptions including multicollinearity and proportional hazards assumptions. All tests were 2-tailed with significance set at *P* = .05. Sensitivity analyses were conducted for logistic and Cox proportional hazards regressions using multiple imputation by chained equations (MICEs), complete case analysis approaches, and false discovery rate adjustments for multiple comparisons. All statistical analyses were conducted using R statistical software, version 4.0.3 in RStudio, version 2023.06.4 + 524 (R Project for Statistical Computing).

## Results

### Patient Demographics and Cancer Characteristics

Of 544 786 AYA patients, 291 899 (median age, 33 years [IQR, 28-37 years]; 186 549 female patients [64%] and 105 350 male patients [36%]) met the inclusion criteria and comprised 1457 American Indian or Alaska Native patients (1%), 8412 Asian patients (3%), 40 851 Black patients (14%), 987 Native Hawaiian or Other Pacific Islander patients (0.3%), and 240 192 White patients (82%) (Table 1). Most patients had private insurance (215 661 [76%]) and lived in metropolitan areas (242 154 [86%]), with higher income (167 546 [63%]) and higher education (162 149 [61%]) levels. Most patients had early-stage or low-grade cancers (206 131 [71%]). Cancers included breast (79 195 [27%]), lymphoma (45 500 [16%]), melanoma (36 724 [13%]), testis (31 413 [11%]), CNS (26 070 [9%]), colon or rectum (22 545 [8%]), cervix (20 923 [7%]), sarcoma (14 951 [5%]), ovary (8982 [3%]), and lung (5596 [2%]). Treatments included surgery (236 222 [81%]), chemotherapy (168 077 [58%]), and radiotherapy (94 750 [32%]).

**Table 1.** Descriptive Statistics of Adolescent and Young Adult Patient Population With Cancer by Race

Characteristic	Adolescent and young adult patients, No. (%)					
	Overall (N = 291 899)	White (n = 240 192)	American Indian or Alaska Native (n = 1457)	Asian (n = 8412)	Black (n = 40 851)	Native Hawaiian or Other Pacific Islander (n = 987)
Deceased	47 613 (16)	35 995 (15)	298 (20)	1319 (16)	9785 (24)	216 (22)
Follow-up, median (IQR), mo	62 (34-102)	64 (36-103)	54 (29-91)	59 (33-96)	55 (29-93)	55 (29-89)
Stage^a^						
I	117 309 (40)	101 820 (42)	497 (34)	2920 (35)	11 787 (29)	285 (29)
II	72 503 (25)	57 335 (24)	361 (25)	2505 (30)	12 008 (29)	294 (30)
III	48 229 (17)	38 177 (16)	292 (20)	1363 (16)	8213 (20)	184 (19)
IV	27 788 (10)	20 606 (9)	168 (12)	961 (11)	5933 (15)	120 (12)
NA (CNS)	26 070 (9)	22 254 (9)	139 (10)	663 (8)	2910 (7)	104 (11)
Stage or grade^a^						
Early or low	206 131 (71)	172 918 (72)	956 (66)	5822 (69)	25 793 (63)	642 (65)
Late or high	85 768 (29)	67 274 (28)	501 (34)	2590 (31)	15 058 (37)	345 (35)
Cancer type						
Breast	79 195 (27)	58 836 (24)	369 (25)	3531 (42)	16 159 (40)	300 (30)
Cervical	20 923 (7)	16 724 (7)	155 (11)	481 (6)	3476 (9)	87 (9)
CNS	26 070 (9)	22 254 (9)	139 (10)	663 (8)	2910 (7)	104 (11)
Colon or rectal	22 545 (8)	17 650 (7)	138 (10)	789 (9)	3873 (10)	95 (10)
Lung	5596 (2)	4391 (2)	17 (1)	270 (3)	902 (2)	16 (2)
Lymphoma	45 500 (16)	35 955 (15)	177 (12)	1257 (15)	7954 (19)	157 (16)
Melanoma	36 724 (13)	36 318 (15)	66 (5)	119 (1)	203 (1)	18 (2)
Ovarian	8982 (3)	7135 (3)	49 (3)	437 (5)	1316 (3)	45 (5)
Sarcoma	14 951 (5)	11 426 (5)	120 (8)	462 (6)	2865 (7)	78 (8)
Testicular	31 413 (11)	29 503 (12)	227 (16)	403 (5)	1193 (3)	87 (9)
Age, median (IQR), y	33 (28-37)	33 (27-37)	33 (26-36)	34 (29-37)	34 (28-37)	33 (27-37)
Sex						
Female	186 549 (64)	148 554 (62)	898 (62)	6225 (74)	30 229 (74)	643 (65)
Male	105 350 (36)	91 638 (38)	559 (38)	2187 (26)	10 622 (26)	344 (35)
Year of diagnosis						
2004-2010	140 225 (48)	116 238 (48)	602 (41)	3734 (44)	19 219 (47)	432 (44)
2011-2017	151 674 (52)	123 954 (52)	855 (59)	4678 (56)	21 632 (53)	555 (56)
Distance to hospital, median (IQR), km	0.19 (0.08-0.45)	0.21 (0.10-0.48)	0.42 (0.13-1.22)	0.11 (0.06-0.23)	0.13 (0.06-0.27)	0.18 (0.08-0.37)
Unknown, No.	24 723	20 766	138	575	3145	99
Income^b^						
Higher income	167 546 (63)	145 048 (66)	544 (41)	6171 (79)	15 119 (40)	664 (75)
Lower income	99 296 (37)	74 109 (34)	775 (59)	1664 (21)	22 526 (60)	222 (25)
Unknown, No.	25 057	21 035	138	577	3206	101
Rurality						
Metropolitan	242 154 (86)	195 702 (85)	864 (62)	7968 (98)	36 728 (92)	892 (92)
Urban-rural	39 091 (14)	35 184 (15)	528 (38)	177 (2)	3126 (8)	76 (8)
Unknown, No.	10 654	9306	65	267	997	19
Education^b^						
More education	162 149 (61)	142 782 (65)	620 (47)	4860 (62)	13 345 (35)	542 (61)
Less education	104 855 (39)	76 502 (35)	700 (53)	2980 (38)	24 328 (65)	345 (39)
Unknown, No.	24 895	20 908	137	572	3178	100
Insurance status						
Private insurance	215 661 (76)	185 251 (79)	667 (47)	6469 (78)	22 617 (57)	657 (68)
Medicaid or Medicare	52 372 (18)	36 663 (16)	664 (47)	1369 (17)	13 408 (34)	268 (28)
Uninsured	17 274 (6)	12 959 (6)	80 (6)	435 (5)	3752 (9)	48 (5)
Unknown, No.	6592	5319	46	139	1074	14
Charlson-Deyo Comorbidity Index						
≤2	288 384 (99)	237 924 (99)	1434 (98)	8336 (99)	39 725 (97)	965 (98)
≥3	3515 (1)	2268 (1)	23 (2)	76 (1)	1126 (3)	22 (2)
Treatment received						
Surgery	236 222 (81)	197 433 (82)	1187 (81)	6717 (80)	30 125 (74)	760 (77)
Chemotherapy	168 077 (58)	132 194 (55)	900 (62)	5666 (67)	28 668 (70)	649 (66)
Radiotherapy	94 750 (32)	74 170 (31)	486 (33)	3508 (42)	16 189 (40)	397 (40)

Abbreviations: CNS, central nervous system; NA, not applicable.

^a^ Stage III or IV disease was considered late stage and World Health Organization grade III or IV were considered high grade for CNS tumors.

^b^ Income and education were dichotomized at the median based on patient zip code area of residence. “Higher income” and “More education” refer to household income and educational attainment above the median, respectively, while “Lower income” and “Less education” refer to income and educational levels below the median, as estimated from 2016 American Community Survey data, adjusted for 2016 inflation.

### Late-Stage Diagnosis

**Figure 1.**
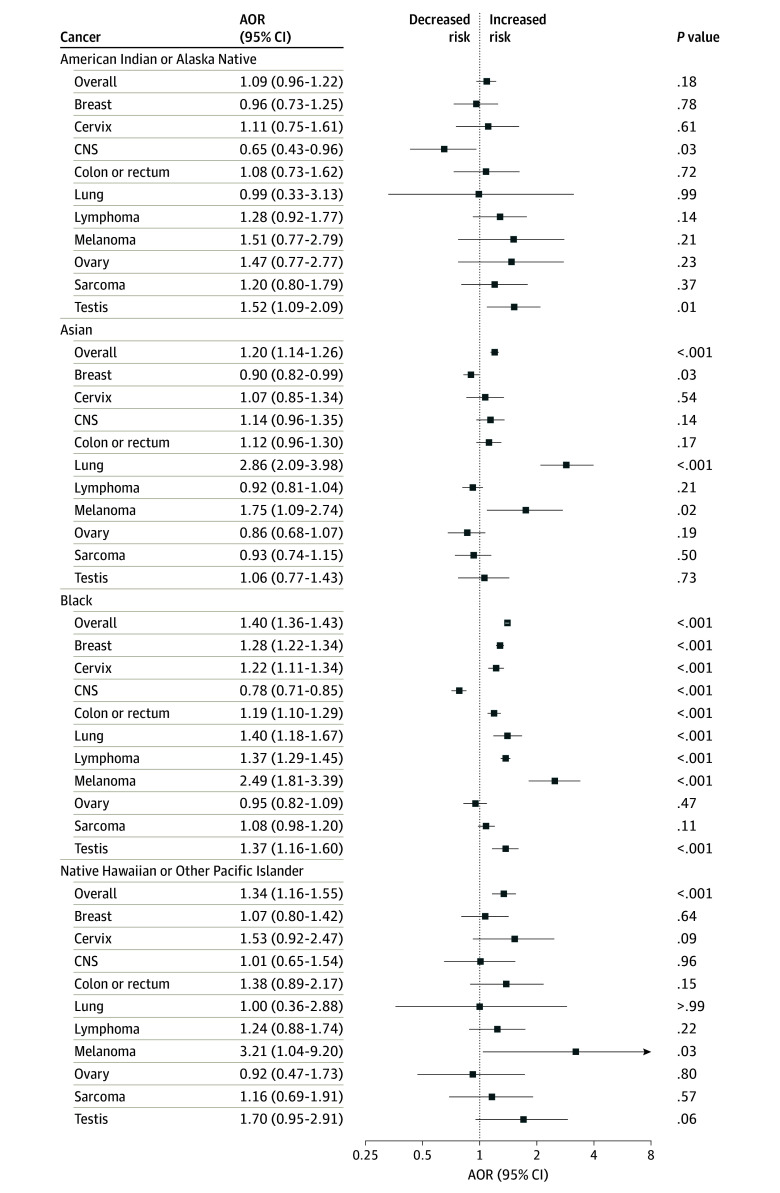
Multivariable Logistic Regression Model for Late Stage at Diagnosis Among Adolescent and Young Adult Patients With Cancer Adjusted odds ratios (AORs) for American Indian or Alaska Native, Asian, Black, and Native Hawaiian or Other Pacific Islander patients are reported compared with White patients (reference group) for the overall cohort and according to cancer site. Late-stage or high-grade were considered either stage III or IV at presentation or World Health Organization grade III or IV (for central nervous system [CNS] tumors). The model was adjusted for sex (except for cancers of the cervix, ovary, and testis), age, income, rurality, education, year of diagnosis, distance to hospital, insurance status, Charlson-Deyo Comorbidity Index, and treatment modalities. Models were assessed for multicollinearity.

After adjustment for sociodemographic and clinical characteristics, there were significantly higher odds of late-stage diagnosis among Asian (AOR, 1.20; 95% CI, 1.14-1.26), Black (AOR, 1.40; 95% CI, 1.36-1.43), and Native Hawaiian or Other Pacific Islander (AOR, 1.34; 95% CI, 1.16-1.55) patients compared with White patients, when comparing all cancer subsites in aggregate (Figure 1).

By disease site, Black patients had significantly higher odds of late-stage diagnosis for virtually all cancers: breast (AOR, 1.28; 95% CI, 1.22-1.34), cervix (AOR, 1.22; 95% CI, 1.11-1.34), colon or rectum (AOR, 1.19; 95% CI, 1.10-1.29), lung (AOR, 1.40; 95% CI, 1.18-1.67), lymphoma (AOR, 1.37; 95% CI, 1.29-1.45), melanoma (AOR, 2.49; 95% CI, 1.81-3.39), and testis (AOR, 1.37; 95% CI, 1.16-1.60), compared with White patients (Figure 1). Black patients with CNS cancer had significantly lower odds of high-grade disease at diagnosis (AOR, 0.78; 95% CI, 0.71-0.85). Asian patients had significantly higher odds of late-stage diagnosis for lung cancer (AOR, 2.86; 95% CI, 2.09-3.98) and melanoma (AOR, 1.75; 95% CI, 1.09-2.74) but lower odds for breast cancer (AOR, 0.90; 95% CI, 0.82-0.99) compared with White patients.

American Indian or Alaska Native patients had significantly higher odds of late-stage diagnosis of testis cancer (AOR, 1.52; 95% CI, 1.09-2.09) and a lower likelihood of high-grade CNS cancer at diagnosis (AOR. 0.65; 95% CI, 0.43-0.96) compared with White patients (Figure 1). Native Hawaiian or Other Pacific Islander patients only had a significantly higher likelihood of having late-stage melanoma at diagnosis (AOR, 3.21; 95% CI, 1.04-9.20) compared with White patients. The risk of late stage at diagnosis was generally consistent on sensitivity analysis with MICE imputation and adjustment for multiplicity, with some attenuation (eFigure 1 in Supplement 1).

### Overall Survival

**Figure 2.**
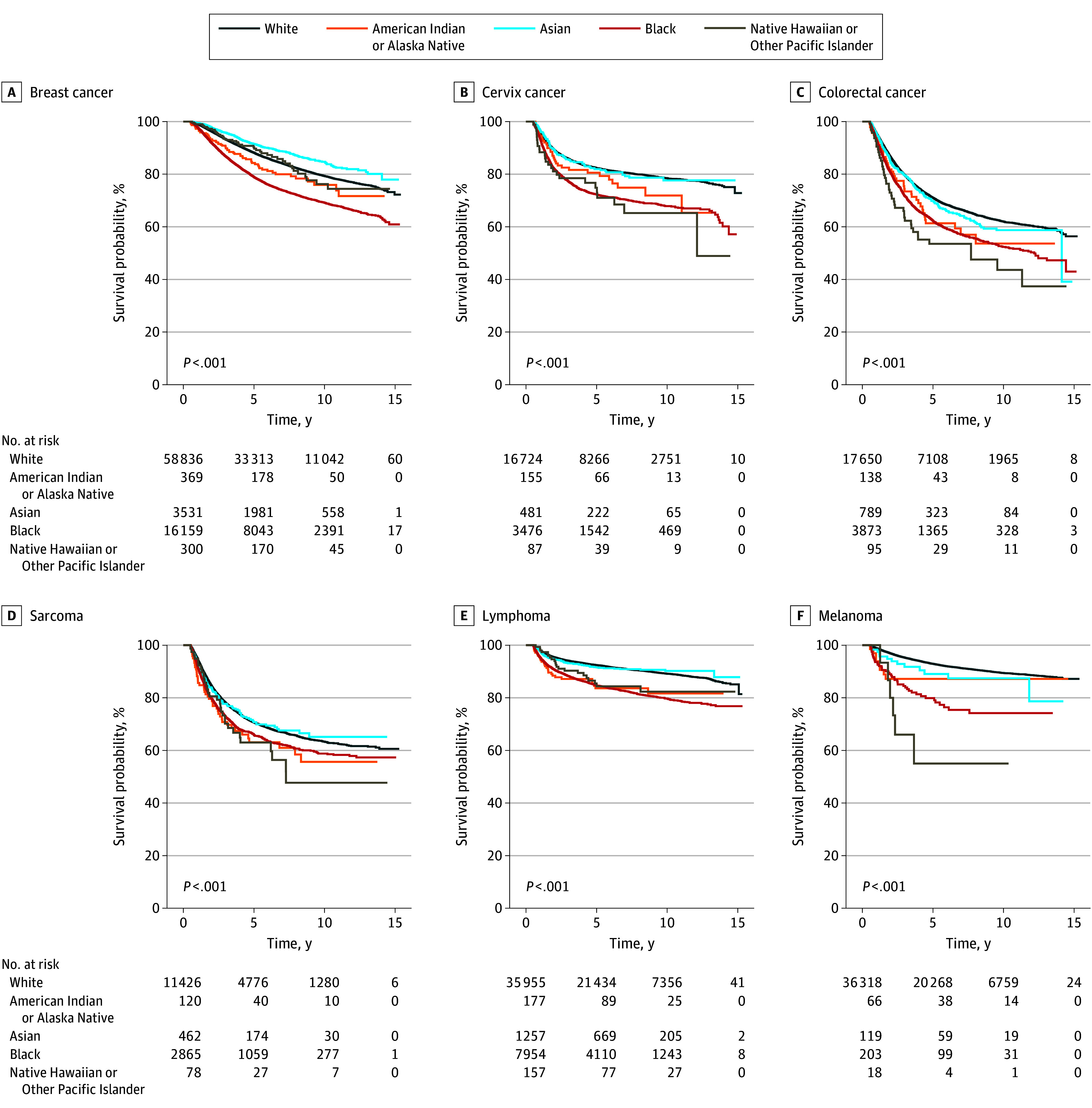
Kaplan-Meier Estimates for Overall Survival Probability for Adolescent and Young Adult Patients by Race Stratified by Cancer *P* values were calculated with log-rank tests.

The median follow-up was 62 months (IQR, 34-102 months) with 47 613 patients (16%) deceased within the study period (Table 1). By race, mortality rates were 20% for American Indian or Alaska Native patients, 16% for Asian patients, 24% for Black patients, 22% for Native Hawaiian or Other Pacific Islander patients, and 15% for White patients. Overall survival varied significantly by race across nearly all cancers, except for cancers of the CNS and ovary (Figure 2; eFigure 2 in Supplement 1).

One-, 5-, and 10-year OS rates are displayed in Table 2. The lowest 10-year OS was observed for American Indian or Alaska Native patients with cancers of the CNS, lung, ovary, and testis and for Native Hawaiian or Other Pacific Islander patients with cervix cancer, colon or rectum cancer, sarcoma, and melanoma. The deficit in 10-year OS rate gaps were measured by the difference between the highest and lowest OS by race. The largest 10-year OS gaps overall occurred among Native Hawaiian or Other Pacific Islander patients with melanoma (difference, 34.4 percentage points; vs White patients), colon or rectum cancer (difference, 18.4 percentage points; vs White patients), and sarcoma (difference, 17.5 percentage points; vs Asian patients).

**Table 2.** One-, 5-, and 10-Year OS Estimates Among AYA Patients With Cancer

Race and cancer	Survival, % (95% CI)			OS rate deficit, percentage point difference^a^
	At 1 y	At 5 y	At 10 y	
Breast				
White	99.1 (99.0-99.2)	88.2 (87.9-88.5)	79.3 (78.9-79.8)	15.5
American Indian or Alaska Native	97.3 (95.6-99.0)	84.3 (80.2-88.5)	75.9 (70.0-82.3)	
Asian	99.5 (99.2-99.7)	91.5 (90.5-92.6)	84.7 (83.0-86.4)^b^	
Black	97.9 (97.7-98.1)	78.9 (78.2-79.6)	69.2 (68.2-70.1)^c^	
Native Hawaiian or Other Pacific Islander	99.0 (97.9-100.0)	90.2 (86.6-94.0)	76.2 (69.2-84.0)	
CNS				
White	97.1 (96.9-97.4)	78.0 (77.4-78.6)	64.1 (63.2-65.0)	8.6
American Indian or Alaska Native	96.4 (93.3-99.5)	73.8 (65.7-82.8)	61.4 (50.4-74.8)^c^	
Asian	97.1 (95.8-98.4)	77.1 (73.5-80.9)	65.5 (60.4-71.1)	
Black	96.2 (95.5-96.9)	79.2 (77.6-80.9)	69.3 (66.9-71.9)	
Native Hawaiian or Other Pacific Islander	94.1 (89.7-98.8)	77.7 (69.1-87.5)	70.0 (59.1-82.9)^b^	
Cervical				
White	96.0 (95.7-96.3)	82.3 (81.7-82.9)	78.5 (77.7-79.2)^b^	13.3
American Indian or Alaska Native	94.7 (91.2-98.3)	80.6 (74.2-87.5)	71.9 (62.8-82.3)	
Asian	96.6 (95.0-98.3)	82.0 (78.3-85.8)	77.7 (73.1-82.5)	
Black	93.5 (92.7-94.3)	72.3 (70.8-73.9)	67.9 (66.0-69.8)	
Native Hawaiian or Other Pacific Islander	88.3 (81.8-95.4)	74.9 (65.7-85.3)	65.2 (53.9-79.0)^c^	
Colon or rectal				
White	95.5 (95.2-95.8)	71.0 (70.3-71.8)	62.0 (61.1-63.0)^b^	18.4
American Indian or Alaska Native	94.2 (90.4-98.2)	61.4 (52.5-71.7)	53.6 (43.0-66.9)	
Asian	95.4 (93.9-96.9)	69.8 (66.3-73.5)	58.7 (54.3-63.6)	
Black	93.0 (92.2-93.8)	62.4 (60.7-64.1)	52.4 (50.2-54.6)	
Native Hawaiian or Other Pacific Islander	90.5 (84.7-96.6)	53.5 (43.7-65.5)	43.6 (31.9-59.5)^c^	
Lung				
White	82.9 (81.8-84.0)	51.1 (49.5-52.7)	43.4 (41.5-45.4)^b^	14.5
American Indian or Alaska Native	87.8 (73.4-100.0)	57.8 (36.6-91.2)	28.9 (6.7-100.0)^c^^,^^d^	
Asian	86.9 (82.9-91.0)	45.4 (39.2-52.5)	29.3 (21.3-40.2)	
Black	79.4 (76.8-82.1)	44.2 (40.8-47.8)	40.0 (36.5-44.0)	
Native Hawaiian or Other Pacific Islander	87.5 (72.7-100.0)	60.9 (40.7-91.1)	36.6 (16.1-83.1)	
Ovarian				
White	97.1 (96.7-97.5)	82.6 (81.6-83.5)	73.3 (71.9-74.7)	17.1
American Indian or Alaska Native	93.8 (87.1-100.0)	76.5 (64.6-90.6)	61.2 (43.0-87.1)^c^	
Asian	95.8 (94.0-97.7)	82.9 (79.0-86.9)	75.3 (70.4-80.6)	
Black	96.1 (95.1-97.2)	83.3 (81.2-85.5)	78.3 (75.6-81.1)^b^	
Native Hawaiian or Other Pacific Islander	91.1 (83.2-99.8)	77.2 (65.7-90.7)	70.6 (57.7-86.5)	
Sarcoma				
White	95.0 (94.7-95.4)	70.7 (69.8-71.6)	63.4 (62.2-64.5)	17.5
American Indian or Alaska Native	88.2 (82.6-94.2)	63.1 (54.2-73.4)	55.7 (45.2-68.6)	
Asian	93.6 (91.4-95.9)	70.7 (66.2-75.6)	65.2 (59.5-71.4)^b^	
Black	92.1 (91.1-93.1)	65.8 (64.0-67.8)	58.8 (56.5-61.2)	
Native Hawaiian or Other Pacific Islander	94.9 (90.1-99.9)	63.0 (52.4-75.7)	47.7 (34.2-66.6)^c^	
Lymphoma				
White	98.0 (97.9-98.2)	92.4 (92.1-92.7)	89.1 (88.7-89.5)	10.5
American Indian or Alaska Native	94.9 (91.6-98.2)	83.6 (77.9-89.7)	81.6 (75.0-88.8)	
Asian	97.7 (96.9-98.6)	91.5 (89.9-93.2)	90.2 (88.2-92.2)^b^	
Black	95.4 (94.9-95.8)	84.7 (83.8-85.5)	79.7 (78.5-80.8)^c^	
Native Hawaiian or Other Pacific Islander	98.7 (96.9-100.0)	84.3 (78.1-91.0)	82.3 (75.2-90.1)	
Testicular				
White	99.3 (99.2-99.4)	96.7 (96.5-96.9)	94.8 (94.5-95.2)^b^	8.3
American Indian or Alaska Native	99.1 (97.9-100.0)	89.3 (85.0-93.8)	86.5 (81.4-92.0)^c^	
Asian	98.7 (97.7-99.8)	94.8 (92.5-97.1)	91.6 (87.8-95.6)	
Black	97.8 (97.0-98.6)	93.4 (91.9-94.9)	90.9 (88.5-93.3)	
Native Hawaiian or Other Pacific Islander	100.0 (100.0-100.0)	92.6 (86.5-99.3)	92.6 (86.5-99.3)	
Melanoma				
White	98.9 (98.7-99.0)	92.9 (92.6-93.2)	89.4 (89.0-89.8)^b^	34.4
American Indian or Alaska Native	93.9 (88.2-99.9)	87.2 (79.3-95.9)	87.2 (79.3-95.9)	
Asian	98.3 (96.0-100.0)	89.0 (83.0-95.5)	87.4 (80.7-94.6)	
Black	93.6 (90.2-97.0)	79.9 (74.2-86.0)	74.2 (67.5-81.6)	
Native Hawaiian or Other Pacific Islander	100.0 (100.0-100.0)	55.0 (32.9-91.9)	55.0 (32.9-91.9)^c^	

Abbreviations: AYA, adolescent and young adult; CNS, central nervous system; OS, overall survival.

^a^ The OS rate deficits were calculated as the difference between the 10-year OS rate of the race categories with the highest and lowest 10-year OS rates, respectively.

^b^ The highest 10-year OS.

^c^ The lowest 10-year OS.

^d^ Extrapolated survival rate due to low event counts at this time point.

By cancer, racial group pairs with the largest gaps between the lowest and highest 10-year OS estimates were Black and Asian patients with breast cancer (69.2% vs 84.7%; difference, 15.5 percentage points), American Indian or Alaska Native and Native Hawaiian or Other Pacific Islander patients with CNS cancer (61.4% vs 70.0%; difference, 8.6 percentage points), Native Hawaiian or Other Pacific Islander and White patients with cervical cancer (65.2% vs 78.5%; difference, 13.3 percentage points), Native Hawaiian or Other Pacific Islander and White patients with colon or rectal cancer (43.6% vs 62.0%; difference, 18.4 percentage points), American Indian or Alaska Native and White patients with lung cancer (28.9% vs 43.4%; difference, 14.5 percentage points), American Indian or Alaska Native and Black patients with ovarian cancer (61.2% vs 78.3%; difference, 17.1 percentage points), Native Hawaiian or Other Pacific Islander and Asian patients with sarcoma (47.7% vs 65.2%; difference, 17.5 percentage points), Black and Asian patients with lymphoma (79.7% vs 90.2%; difference, 10.5 percentage points), American Indian or Alaska Native and White patients with testicular cancer (86.5% vs 94.8%; difference, 8.3 percentage points), and Native Hawaiian or Other Pacific Islander and White patients with melanoma (55.0% vs 89.4%; difference, 34.4 percentage points) (Table 2).

**Figure 3.**
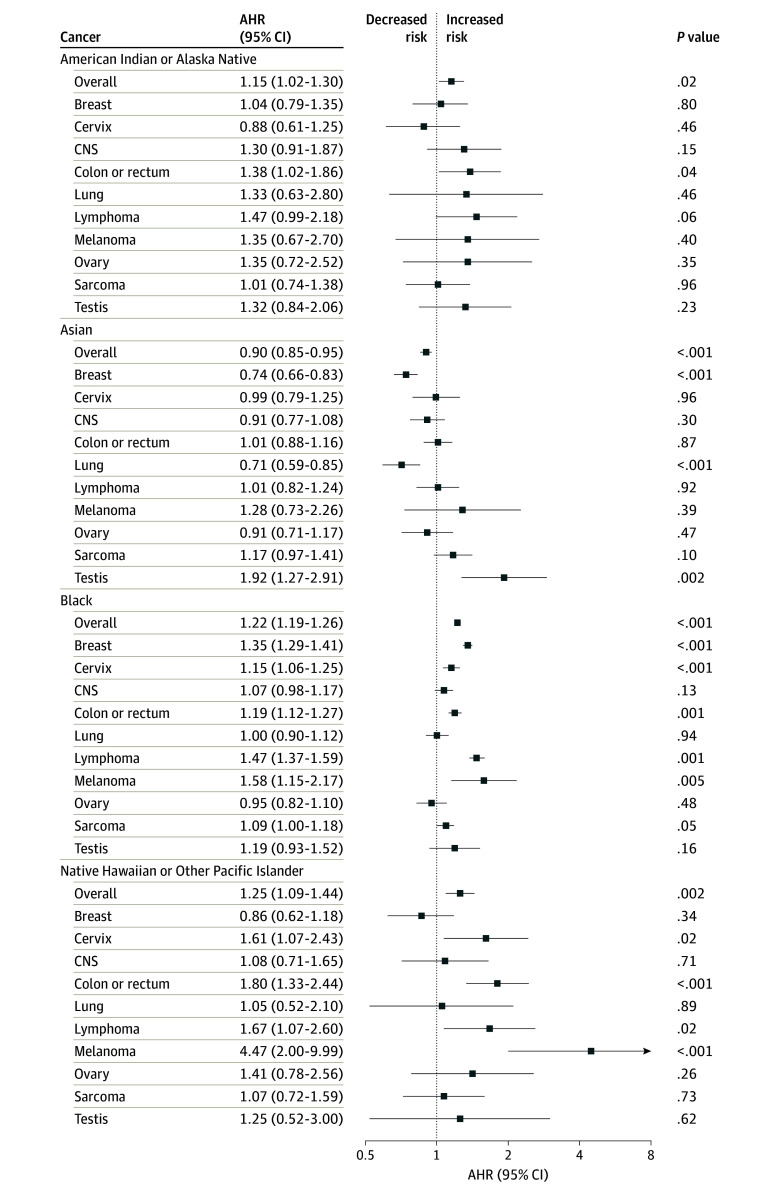
Multivariable Cox Proportional Hazards Regression for Risk of Death Among Adolescent and Young Adult Patients With Cancer The risk of death for American Indian or Alaska Native, Asian, Black, and Native Hawaiian or Other Pacific Islander patients is reported compared with White patients (reference group) and was adjusted for sex (except for cancers of the cervix, ovary, and testis), age, income, rurality, education, year of diagnosis, distance to hospital, insurance status, Charlson-Deyo comorbidity index, and treatment modalities. Proportional hazards assumptions were evaluated with covariates violating the assumptions fit into the regression model with stratification. AHR indicates adjusted hazard ratio; CNS, central nervous system.

After adjustment for sociodemographic and cancer covariates across all cancers, the risk of death was significantly higher among American Indian or Alaska Native (AHR, 1.15; 95% CI, 1.02-1.30), Black (AHR, 1.22; 95% CI, 1.19-1.26), and Native Hawaiian or Other Pacific Islander (AHR, 1.25; 95% CI, 1.09-1.44) patients compared with White patients (Figure 3). Asian patients had a significantly lower risk of death (AHR, 0.90; 95% CI, 0.85-0.95) compared with White patients.

Stratified by cancer, Native Hawaiian or Other Pacific Islander patients had significantly higher risk of death for melanoma (AHR, 4.47; 95% CI, 2.00-9.99), colon or rectum cancer (AHR, 1.80; 95% CI, 1.33-2.44), lymphoma (AHR, 1.67; 95% CI, 1.07-2.60), and cervix cancer (AHR, 1.61; 95% CI, 1.07-2.43) compared with White patients. Black patients had a significantly higher risk of death for melanoma (AHR, 1.58; 95% CI, 1.15-2.17), lymphoma (AHR, 1.47; 95% CI, 1.37-1.59), breast cancer (AHR, 1.35; 95% CI, 1.29-1.41), cervix cancer (AHR, 1.15; 95% CI, 1.06-1.25), colon or rectum cancer (AHR, 1.19; 95% CI, 1.12-1.27), and sarcoma (AHR, 1.09; 95% CI, 1.00-1.18). American Indian or Alaska Native patients had a significantly higher risk of death for colon or rectum cancer (AHR, 1.38; 95% CI, 1.02-1.86). Asian patients had a significantly lower risk of death for breast cancer (AHR, 0.74; 95% CI, 0.66-0.83) and lung cancer (AHR, 0.71; 95% CI, 0.59-0.85). On late-stage subset analysis, only Native Hawaiian or Other Pacific Islander (AHR, 1.34; 95% CI, 1.16-1.55) and Black (AHR, 1.40; 95% CI, 1.36-1.43) patients had a higher risk of death compared with White patients. The significance for risk of death was generally consistent on sensitivity analysis with MICE imputation and adjustment for multiplicity, with some attenuation (eFigure 3 in Supplement 1).

## Discussion

This analysis of AYA patients with cancer in the US revealed significant racial disparities in late-stage diagnosis and OS. Black patients had higher odds of late-stage diagnosis compared with White patients and a significantly higher risk of death for most cancers. Native Hawaiian or Other Pacific Islander and American Indian or Alaska Native patients had the greatest deficits in 10-year OS rates among the greatest number of cancers, despite not having a corresponding increased risk of late stage at diagnosis. Asian patients generally had a significantly lower risk of death compared with White patients, despite having a higher risk of late stage at diagnosis overall. These findings not only broaden the understanding of racial disparities in cancer epidemiology in understudied AYA populations with cancer but also underscore the need for increased research focusing on Indigenous AYA patients, who are often excluded from national studies.^16,17,24^

### AYA Cancer Racial Disparities in Late-Stage Diagnosis and OS

Research on AYA cancer is limited, especially regarding racial disparities. Many studies neglect the federal guidelines established in 1997 by not including all 5 racial categories when evaluating racial disparities, promoting Indigenous erasure through omission of data on Native Hawaiian or Other Pacific Islander and American Indian or Alaska Native patients.^17,20^ This study is likely the most inclusive and comprehensive evaluation of AYA cancer survival and stage at diagnosis disparities across all federally defined racial categories.

Black AYA patients were significantly more likely to receive a diagnosis of late-stage cancer in 7 of 10 cancers studied compared with White patients, with a significantly higher risk of death for breast cancer, cervix cancer, colon or rectum cancer, lymphoma, and melanoma. These findings are consistent with previous studies.^3,12,14,15,25,26,27,28^ In contrast, while American Indian or Alaska Native and Native Hawaiian or Other Pacific Islander patients also exhibited a significantly higher risk of death compared with White patients, the pattern of late-stage diagnosis differed markedly from Black patients. Indigenous AYA patients had a higher risk of late-stage diagnosis in only 1 of the 10 cancers each compared with White patients. This finding suggests that factors other than late-stage diagnosis at presentation may be associated with poorer survival outcomes among Indigenous patients. Despite Black, Asian, and Native Hawaiian or Other Pacific Islander patients being more likely to receive a diagnosis of late-stage disease, only Black and Native Hawaiian or Other Pacific Islander patients with late-stage disease had higher risks of death compared with White patients.

Achieving cancer care equity for all races and ethnicities requires further examination of the root causes of these disparities.^29^ Factors not explicitly explored in this study that may also affect cancer outcomes include inadequate transportation access, leading to treatment delays, as well as poor perceived quality of care, leading to mistrust in physicians, among others.^29^ Along with the social determinants of health, discrimination, and lack of patient trust in health care professionals, these factors may all be associated with poor cancer outcomes for certain groups.^30^

### Cancer-Specific Racial Disparities in OS Among AYA Patients

Given AYA patients’ long survivorship potential,^23^ understanding long-term survival gaps helps identify the largest disparities. The 10-year OS gap was lowest for testicular cancer at 8 percentage points, likely due to the overall favorable outcomes nearing 90% survival across all 5 racial categories. In stark contrast, this gap was more than 4 times higher between White (89%) and Native Hawaiian or Other Pacific Islander (55%) patients with melanoma, reflecting a similar trend in adjusted survival models.

Black and Native Hawaiian or Other Pacific Islander patients with melanoma had a significantly increased risk of death and late-stage diagnosis. Unlike Black patients, Native Hawaiian or Other Pacific Islander patients were significantly more likely to receive a diagnosis of late-stage melanoma. Melanoma was also the only cancer among the 10 cancers studied that demonstrated a higher likelihood of late-stage diagnosis among the Native Hawaiian or Pacific Islander population. Prior studies demonstrated a higher incidence of melanoma among lighter-skinned populations but higher mortality and late-stage diagnosis among darker-skinned groups, consistent with the present findings.^31,32,33,34,35^ Few, if any, studies ever included Indigenous populations, including Native Hawaiian or Other Pacific Islander and American Indian or Alaska Native populations as federally defined.

Inconspicuous oral melanomas have lower screening detection and are more common among Hispanic and Black populations.^31,32^ Moreover, physicians and patients may carry false expectations about melanoma risk among darker-skinned populations, which together may be associated with lower screening rates and worse outcomes.^34,35^ Survival may also be associated with the histologic subtype of melanoma, as lighter-skinned individuals are prone to cutaneous melanoma vs acral, mucosal, or ocular subtypes, with the former being more responsive to immunotherapy, indicating a need for further study and an emphasis on inclusivity in ongoing clinical trials.^18,36,37,38^

Prior studies have shown that Asian patients have higher rates of late-stage cancer diagnoses, including melanoma and lung cancers, compared with White patients.^33,39,40^ Despite this finding, Asian patients with late-stage disease do not have significantly lower OS, potentially due to better responses to immunotherapy or radiotherapy, higher socioeconomic status, improved health care access, lower exposure to environmental carcinogens, and genetic factors such as interethnic variants in the *EGFR* gene.^26,41,42^ Overall, late stage at diagnosis is 1 benchmark, but it may not always correlate with end points such as survival. Asian patients have lower OS for testicular cancer, a finding not shared by Native Hawaiian or Other Pacific Islander patients, underscoring the need to consider Asian and Native Hawaiian or Other Pacific Islander populations separately.

### Combating Indigenous Erasure Through Data Inclusion, Disaggregation, and Federally Defined Race Standards

Most AYA cancer research investigating racial disparities in the US excludes Indigenous American Indian or Alaska Native or Native Hawaiian or Other Pacific Islander populations.^10,14,43,44^ This is concerning given that Native Hawaiian or Other Pacific Islander and American Indian or Alaska Native patients have inferior OS for many types of cancers. Although a significant proportion of disparities research focuses on Black or Hispanic patients, less is understood about American Indian or Alaska Native and Native Hawaiian or Other Pacific Islander patients. In the present study, both Black and Native Hawaiian or Other Pacific Islander patients had significantly worse survival compared with White AYA patients overall, and the 2 Indigenous AYA populations had among the widest survival gaps by cancer. American Indian or Alaska Native patients had the worst 10-year OS for cancers of the CNS, lung, ovary, and testis, while Native Hawaiian or Other Pacific Islander patients had the worst 10-year OS for cancers of the cervix and colon or rectum, as well as sarcoma and melanoma. Wider 95% CIs due to small sample sizes for the American Indian or Alaska Native and Native Hawaiian or Other Pacific Islander populations may have affected these results. Nonetheless, it is important to include these Indigenous groups disaggregated as federally defined so they can be assessed, mitigating the erasure of significant health disparities faced by these populations.^16,17,45^

The issue of Indigenous erasure for the Native Hawaiian or Other Pacific Islander population is further compounded by inappropriate aggregation with Asian patients. Despite being 2 distinct federally recognized races since the 1997 Office of Management and Budget race standards were established, Native Hawaiian or Other Pacific Islander patient data are often aggregated with other unrelated populations,^28,44,46,47,48,49^ misclassified as Asian,^26,50^ or omitted altogether.^51^ This is despite federal standards and policies mandating the reporting of all 5 federally defined racial categories in health data since the passage of the Patient Protection and Affordable Care Act.^20,52^ Multiple cancer studies in the adult population have demonstrated the importance of disaggregation between these 2 racial populations.^16,17,21,24,53,54,55,56,57,58,59,60,61,62^

Multiple studies inappropriately aggregating Asian and Native Hawaiian or Other Pacific Islander AYA patients have suggested similar or superior survival outcomes of the aggregated group compared with White patients.^28,44,46,47,48,49^ However, our results show that Native Hawaiian or Other Pacific Islander AYA patients have significantly lower OS than White patients, while Asian patients have shown higher OS for most cancers. This finding highlights the importance of disaggregating Asian and Native Hawaiian or Other Pacific Islander patient data to avoid masking health disparities. Future AYA studies must disaggregate Asian and Native Hawaiian or Other Pacific Islander populations to address both structural racism and improve care for these patients.^16,17,45^ Given that patient-physician race concordance with culturally competent care has the potential to improve quality of care for Indigenous patients, these results emphasize the importance of building a more robust American Indian or Alaska Native and Native Hawaiian or Other Pacific Islander physician workforce for AYA patients.^63,64,65,66^

### Strengths and Limitations

This study has some strengths, including its large sample size and comprehensive evaluation of the 10 deadliest cancers diagnosed in AYA patients, including all 5 federally defined racial categories. To our knowledge, this is the first study to comprehensively evaluate these AYA cancers in the American Indian or Alaska Native and Native Hawaiian or Other Pacific Islander populations, which affirms the continued need to combat structural racism and Indigenous data erasure.

This study also has some limitations, including its retrospective nature and reliance on the hospital-based NCDB, which lacks clinically important end points (eg, patient-reported outcomes, causes of death to compute competing risks analyses, and disease-free survival) and psychosocial metrics (eg, perceived quality of care, health care professional biases, and culturally competent care). The study also lacks data on secondary cancers and recurrence rates, which would add valuable context. In addition, while White patients were used as the reference group due to their majority status, the goal is equitable outcomes for all, not implying White patients as the criterion standard. Furthermore, we did not examine differences by Hispanic ethnicity, given the smaller sample sizes of Indigenous populations.

## Conclusions

This is likely the most inclusive and comprehensive study to evaluate cancer disparities in stage at diagnosis and survival among AYA patients across all 5 federally defined racial categories. Adolescent and young adult patients with cancer vary in survival and stage at diagnosis by race, with some of the largest gaps seen in melanoma, colorectal cancer, and sarcoma. American Indian or Alaska Native, Black, and Native Hawaiian or Other Pacific Islander AYA patients are disproportionately affected by multiple cancers, with significantly inferior OS. Indigenous data inclusion has uncovered racial disparities previously masked by data omission and aggregation, emphasizing the need for consistent inclusion of these federally defined racial categories.

## Notes

Supplement 1. **eFigure 1.** Multivariable Logistic Regression for Late-Stage at Diagnosis Among AYA Patients With Cancer **eFigure 2.** Kaplan-Meier Estimates for Overall Survival Probability for AYA Patients by Race Stratified by Cancer **eFigure 3.** Multivariable Cox Proportional Hazards Regression for Risk of Death for AYA Patients With Cancer

Supplement 2.
Data Sharing Statement
