# Supplementary material for: Racial Disparities in Cancer Stage at Diagnosis and Survival for Adolescents and Young Adults
Source: JAMA Netw Open. 2024 Aug 30;7(8):e2430975. doi: 10.1001/jamanetworkopen.2024.30975 (PMC11365006; doi:10.1001/jamanetworkopen.2024.30975)
Supplement: Supplement 1. — eFigure 1. Multivariable Logistic Regression for Late-Stage at Diagnosis Among AYA Patients With Cancer eFigure 2. Kaplan-Meier Estimates for Overall Survival Probability for AYA Patients by Race Stratified by Cancer eFigure 3. Multivariable Cox Proportional Hazards Regression for Risk of Death for AYA Patients With Cancer [file jamanetwopen-e2430975-s001.pdf]

Supplementary Online Content

Taparra K, Kekumano K, Benavente R, et al. Racial disparities in cancer stage at diagnosis and survival for adolescents and young adults. *JAMA Netw Open.* 2024;7(8):e2430975. doi:10.1001/jamanetworkopen.2024.30975

eFigure 1. Multivariable Logistic Regression for Late-Stage at Diagnosis Among AYA Patients With Cancer
eFigure 2. Kaplan-Meier Estimates for Overall Survival Probability for AYA Patients by Race Stratified by Cancer
eFigure 3. Multivariable Cox Proportional Hazards Regression for Risk of Death for AYA Patients With Cancer

This supplementary material has been provided by the authors to give readers additional information about their work.

© 2024 Taparra K et al. *JAMA Network Open.*

Cancer	aOR	95%CI	Forest Plot	P	P_{FDR}
**AI/AN**					
Overall	1.10	(0.98-1.23)		0.095	0.237
Breast	0.98	(0.77-1.25)		0.867	1.000
Cervical	0.91	(0.60-1.40)		0.677	1.000
CNS	0.69	(0.48-1.00)		0.053	0.266
Colorectal	1.03	(0.73-1.45)		0.869	1.000
Lung	1.33	(0.47-3.74)		0.590	1.000
Lymphoma	1.24	(0.92-1.68)		0.156	0.782
Melanoma	1.36	(0.76-2.42)		0.300	0.750
Ovarian	2.32	(1.22-4.42)		**0.011**	0.056
Sarcoma	1.24	(0.85-1.80)		0.261	0.870
Testicular	1.69	(1.16-2.44)		**0.006**	**0.028**
**Asian**					
Overall	1.21	(1.15-1.27)		**<0.001**	**<0.001**
Breast	0.92	(0.85-1.01)		0.069	0.347
Cervical	1.11	(0.86-1.44)		0.424	1.000
CNS	1.12	(0.95-1.31)		0.182	0.606
Colorectal	1.10	(0.95-1.27)		0.214	0.714
Lung	3.18	(2.34-4.33)		**<0.001**	**<0.001**
Lymphoma	0.93	(0.83-1.05)		0.264	0.880
Melanoma	1.58	(1.02-2.44)		**0.039**	0.193
Ovarian	0.74	(0.59-0.93)		**0.011**	0.056
Sarcoma	0.95	(0.77-1.17)		0.623	1.000
Testicular	1.20	(0.86-1.69)		0.288	0.960
**Black**					
Overall	1.38	(1.34-1.41)		**<0.001**	**<0.001**
Breast	1.28	(1.23-1.34)		**<0.001**	**<0.001**
Cervical	0.88	(0.79-0.97)		**0.013**	0.132
CNS	0.76	(0.70-0.83)		**<0.001**	**<0.001**
Colorectal	1.16	(1.07-1.25)		**<0.001**	**0.002**
Lung	1.42	(1.21-1.68)		**<0.001**	**<0.001**
Lymphoma	1.34	(1.27-1.41)		**<0.001**	**<0.001**
Melanoma	2.40	(1.78-3.24)		**<0.001**	**<0.001**
Ovarian	0.90	(0.78-1.04)		0.157	0.522
Sarcoma	1.08	(0.99-1.19)		0.094	0.797
Testicular	1.38	(1.15-1.65)		**<0.001**	**0.006**
**NHPI**					
Overall	1.32	(1.16-1.51)		**<0.001**	**<0.001**
Breast	1.09	(0.83-1.42)		0.550	1.000
Cervical	1.16	(0.67-2.02)		0.598	1.000
CNS	1.00	(0.67-1.50)		0.984	1.000
Colorectal	1.38	(0.90-2.10)		0.140	0.700
Lung	1.03	(0.37-2.86)		0.951	1.000
Lymphoma	1.15	(0.83-1.59)		0.395	0.987
Melanoma	2.03	(0.73-5.62)		0.174	0.581
Ovarian	0.88	(0.46-1.68)		0.696	1.000
Sarcoma	1.39	(0.88-2.20)		0.159	0.797
Testicular	1.18	(0.64-2.19)		0.600	1.000

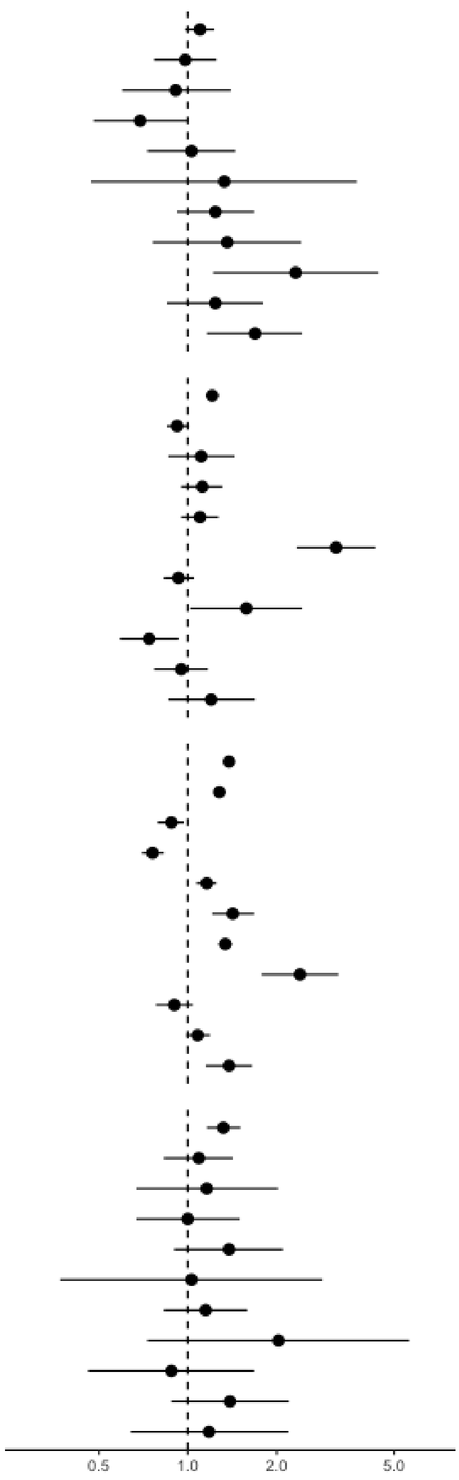

eFigure 1. Multivariable Logistic Regression for Late-Stage at Diagnosis Among AYA Patients With Cancer. The model included data imputed using the Multiple Imputation by Chained Equations (MICE) method. Adjusted p-values were calculated using the False Discovery Rate (FDR) method to account for multiple comparisons (P_{FDR}). Adjusted odds ratios for AI/AN, Asian, Black, and NHPI patients are reported compared to White patients (reference group) for the overall cohort and according to cancer site. Late stage/high grade were considered either stage III/IV at presentation or WHO grade III/IV (for

© 2024 Taparra K et al. *JAMA Network Open.*

CNS tumors). The model was adjusted for sex (except for cervical, ovarian, and testicular cancers), age, income, rurality, education, year of diagnosis, distance to hospital, insurance status, Charlson Deyo comorbidity index, and treatment modalities. Models were assessed for multicollinearity. Abbreviations: aOR=Adjusted Odds Ratio; AI/AN=American Indian or Alaska Native; NHPI=Native Hawaiian or other Pacific Islander.

© 2024 Taparra K et al. *JAMA Network Open.*

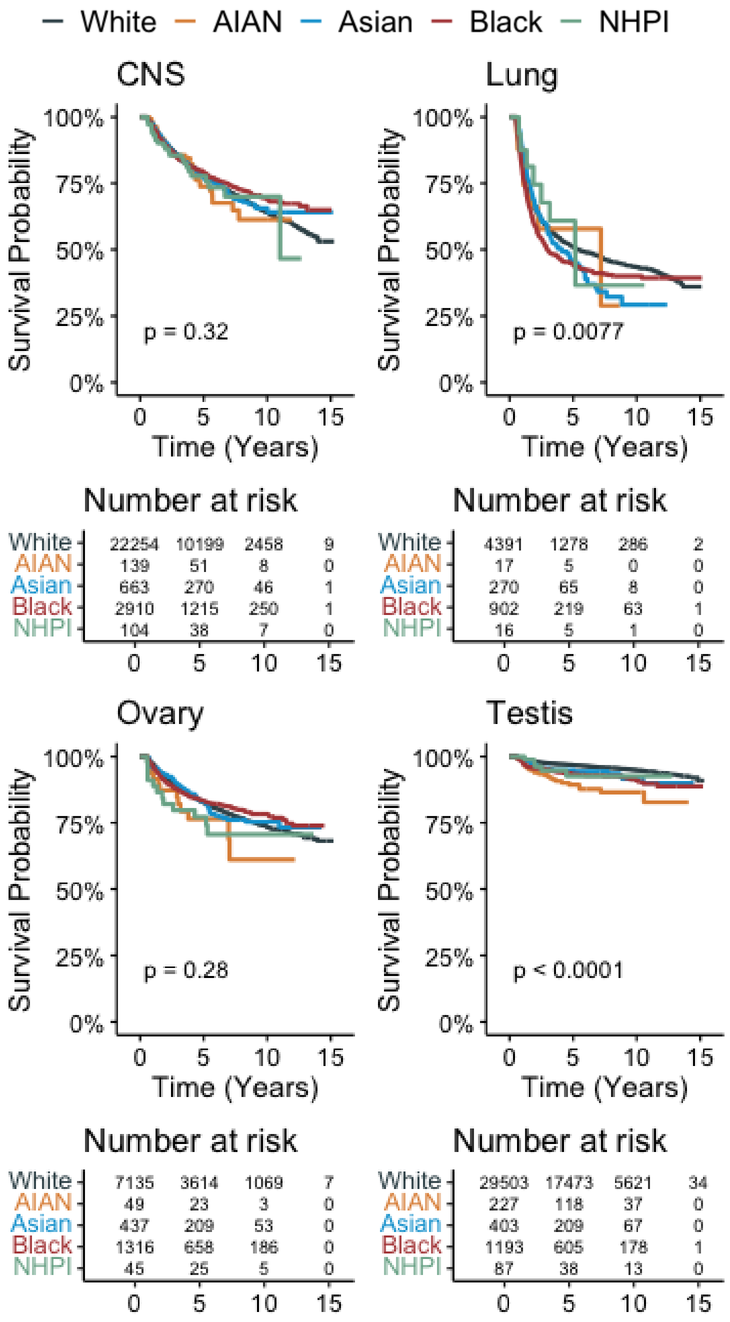

eFigure 2. Kaplan-Meier Estimates for Overall Survival Probability for AYA Patients by Race Stratified by Cancer. P-values were calculated with log-rank tests. Tables below each Kaplan-Meier plot show the number of patients at risk of death at time points 0-, 5-, 10-, and 15-years. Abbreviations: CNS=Central Nervous System; AI/AN=American Indian or Alaska Native; NHPI=Native Hawaiian or other Pacific Islander.

© 2024 Taparra K et al. *JAMA Network Open.*

Cancer	aHR	95%CI	Forest Plot	P	P_{FDR}
**AI/AN**					
Overall	1.13	(1.01-1.27)		**0.033**	0.083
Breast	1.06	(0.83-1.36)		0.654	1.000
Cervical	0.85	(0.60-1.20)		0.367	1.000
CNS	1.33	(0.95-1.85)		0.095	0.497
Colorectal	1.16	(0.87-1.56)		0.312	1.000
Lung	1.08	(0.51-2.28)		0.840	1.000
Lymphoma	1.52	(1.04-2.22)		**0.032**	0.107
Melanoma	1.00	(0.50-2.01)		0.997	1.000
Ovarian	1.17	(0.66-2.07)		0.596	1.000
Sarcoma	1.03	(0.76-1.40)		0.862	1.000
Testicular	1.55	(1.04-2.32)		**0.033**	0.164
**Asian**					
Overall	0.91	(0.86-0.96)		**<0.001**	**0.002**
Breast	0.73	(0.66-0.81)		**<0.001**	**<0.001**
Cervical	1.04	(0.83-1.29)		0.737	1.000
CNS	0.96	(0.82-1.14)		0.663	1.000
Colorectal	1.03	(0.90-1.17)		0.663	1.000
Lung	0.72	(0.60-0.85)		**<0.001**	**0.002**
Lymphoma	1.04	(0.85-1.27)		0.704	1.000
Melanoma	1.31	(0.76-2.27)		0.330	1.000
Ovarian	0.95	(0.76-1.20)		0.691	1.000
Sarcoma	1.14	(0.95-1.36)		0.168	0.840
Testicular	1.78	(1.18-2.67)		**0.006**	0.056
**Black**					
Overall	1.40	(1.36-1.43)		**<0.001**	**<0.001**
Breast	1.36	(1.30-1.42)		**<0.001**	**<0.001**
Cervical	1.14	(1.05-1.23)		**0.001**	**0.014**
CNS	1.08	(0.99-1.17)		0.099	0.497
Colorectal	1.21	(1.14-1.29)		**<0.001**	**<0.001**
Lung	1.02	(0.92-1.14)		0.682	1.000
Lymphoma	1.48	(1.38-1.59)		**<0.001**	**<0.001**
Melanoma	1.61	(1.19-2.19)		**0.002**	**0.010**
Ovarian	0.93	(0.80-1.07)		0.300	1.000
Sarcoma	1.13	(1.05-1.22)		**0.002**	**0.019**
Testicular	1.23	(0.98-1.55)		0.080	0.268
**NHPI**					
Overall	1.34	(1.16-1.55)		**<0.001**	**0.002**
Breast	0.92	(0.68-1.25)		0.590	1.000
Cervical	1.52	(1.03-2.26)		**0.036**	*0.181*
CNS	1.03	(0.68-1.55)		0.892	1.000
Colorectal	1.78	(1.32-2.40)		**<0.001**	**<0.001**
Lung	1.11	(0.55-2.22)		0.778	1.000
Lymphoma	1.73	(1.12-2.65)		**0.013**	*0.064*
Melanoma	4.46	(2.00-9.96)		**<0.001**	**0.003**
Ovarian	1.56	(0.88-2.76)		0.128	1.000
Sarcoma	1.16	(0.81-1.67)		0.414	1.000
Testicular	1.25	(0.52-3.02)		0.618	1.000

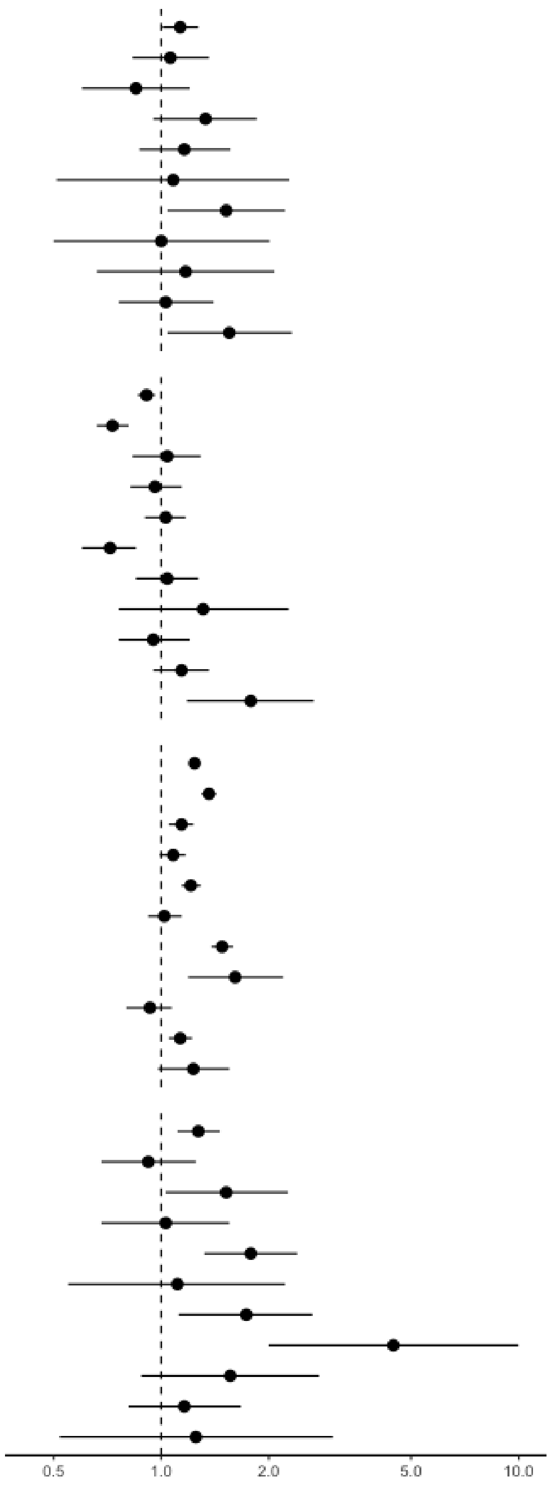

eFigure 3. Multivariable Cox Proportional Hazards Regression for Risk of Death for AYA Patients With Cancer. The model included data imputed using the Multiple Imputation by Chained Equations (MICE) method. Adjusted p-values were calculated using the False Discovery Rate (FDR) method to account for multiple comparisons (P_{FDR}). The risk of death for AI/AN, Asian, Black, and NHPI patients were compared to White patients (reference group) and were adjusted for sex (except for cervical, ovarian, and testicular cancers), age, income, rurality, education, year of diagnosis, distance to hospital,

© 2024 Taparra K et al. *JAMA Network Open.*

insurance status, Charlson Deyo comorbidity index, and treatment modalities. Proportional hazard assumptions were evaluated with covariates violating the assumptions fit into the regression model with stratification. Abbreviations: aHR=Adjusted Hazard Ratio; AI/AN=American Indian or Alaska Native; NHPI=Native Hawaiian or other Pacific Islander; CNS=Central Nervous System.

© 2024 Taparra K et al. *JAMA Network Open.*
