# Supplementary material for: Racial Disparities in Cancer Stage at Diagnosis and Survival for Adolescents and Young Adults
Source: JAMA Netw Open. 2024 Aug 30;7(8):e2430975. doi: 10.1001/jamanetworkopen.2024.30975 (PMC11365006; doi:10.1001/jamanetworkopen.2024.30975)
Supplement: Supplement 2. — Data Sharing Statement [file jamanetwopen-e2430975-s002.pdf]

# Data Sharing Statement

Taparra. Racial Disparities in Cancer Stage at Diagnosis and Survival for Adolescents and Young Adults. *JAMA Netw Open*. Published August 30, 2024.
doi:10.1001/jamanetworkopen.2024.30975

**Data**
**Data available:** No

**Additional Information**
**Explanation for why data not available:** While we are unable to share the data directly, the data is available through the National Cancer Database
